# Bonding Strength of Various Luting Agents between Zirconium Dioxide Crowns and Titanium Bonding Bases after Long-Term Artificial Chewing

**DOI:** 10.3390/ma16237314

**Published:** 2023-11-24

**Authors:** Aimen Bagegni, Justus Borchers, Samuel Beisel, Sebastian B. M. Patzelt, Kirstin Vach, Ralf Kohal

**Affiliations:** 1 Department of Prosthetic Dentistry, Center for Dental Medicine, Medical Center—University of Freiburg, Faculty of Medicine, University of Freiburg, Hugstetter Street 55, 79106 Freiburg, Germany; 2 Mydent Medical Care Center, Krefelder Street 15-17, 41460 Neuss, Germany; 3 Department of Sustainable Systems Engineering, Walter-and-Ingeborg-Herrmann-Chair for Power Ultrasonics and Engineering of Functional Materials, Faculty of Engineering, University of Freiburg, Emmy-Noether-Street 2, 79110 Freiburg, Germany; 4 Private Dental Clinic, Am Dorfplatz 3, 78658 Zimmern ob Rottweil, Germany; 5 Institute of Medical Biometry and Statistics, Medical Center—University of Freiburg, Faculty of Medicine, University of Freiburg, Stefan-Meier-Street 26, 79104 Freiburg, Germany; kirstin.vach@uniklinik-freiburg.de

**Keywords:** implant–abutment connection, hybrid abutment crown, adhesive cement, zirconium dioxide, bonding strength

## Abstract

The use of hybrid abutment crowns bonded extraorally to a titanium bonding base has aesthetic and biological benefits for the prosthetic rehabilitation of oral implants. The objective of this study was to evaluate the effects of luting agents between a zirconium dioxide crown and the titanium bonding base on crown/abutment retention and the subsequent durability of the prosthetic superstructure. Fifty-six implant abutment samples, all restored with a lower first premolar zirconium dioxide crown, were used and divided into seven groups (*n* = 8/group) according to the type of luting agent used: group 1, SpeedCEM Plus; group 2, Panavia SA Cement Universal; group 3, Panavia V5; group 4, RelyX Unicem 2 Automix; group 5, VITA ADIVA IA-Cem; group 6, Ketac CEM; and group 7, Hoffmann’s Phosphate Cement. All specimens were subjected to thermomechanical loading (load of 49 N, 5 million chewing cycles and 54.825 thermocycles in water with temperatures of 5 °C and 55 °C). The surviving samples were exposed to a pull-off force until crown debonding from the bonding base. Overall, 55 samples survived the thermomechanical load. Group 2 showed the highest mean pull-off force value (762 N), whereas group 6 showed the lowest mean value (55 N). The differences between the seven groups were statistically significant (ANOVA, *p* < 0.001). The debonding failure pattern was mainly adhesive and was noticed predominantly at the zirconium dioxide–luting agent interface. Within the scope of the present investigation, it was shown that most of the luting agents are suitable for “cementation” of a zirconium dioxide crown onto a titanium base since the debonding forces are above a recommended value (159 N).

## 1. Introduction

Ceramic crowns on osseointegrated oral titanium implants have been well documented as a primary treatment option for the replacement of a missing single tooth and have presented high survival rates [1,2,3]. After osseointegration, resistance against fatigue and long-term stability of the implant–abutment complex become important factors for clinical success. Generally, the prosthetic superstructure can be attached to the oral implant using two different methods. In the first method, the crown can be cemented intraorally onto the already inserted implant abutment. In the second method, the abutment (titanium bonding base) and the crown are joined with a luting agent extraorally and then attached intraorally to the implant via screw retention. The advantages of the second method are that the screw-retained superstructure can be easily removed (for repair or adjustment) and that the crown–abutment interface can be placed beyond the gingival sulcus (superior aesthetic). Finally, the possibility of cement residues being trapped in peri-implant tissues is excluded [4,5]. Closing the screw access canal with a composite material remains the downside of this method, as this is not suitable for every clinical situation, e.g., at the buccal aspect of the aesthetic area of the upper jaw [6].

Titanium oral implants are the gold standard in dentistry for implant-supported oral restorations [7]. On the abutment level, titanium abutments show higher fracture strengths than pure ceramic abutments [8]. Furthermore, a comparative in vitro study was able to show that the wear during artificial loading on the titanium implant is significantly lower when it is connected to a titanium abutment compared to when it is connected to an all-ceramic zirconium dioxide abutment [9]. For these reasons, dental ceramics are therefore only used as a component for individual hybrid metal–ceramic abutments, hybrid abutment crowns or all-ceramic crowns production [10].

Individual hybrid abutments made of a metal core and ceramic veneering can compensate for an unfavorable implant axis, are able to shift the cementation margin to or above the gingival level for better biocompatibility, and can form an emergence profile that is aesthetically pleasing [11]. The ceramic part of the abutment, usually made of zirconium dioxide, is bonded extraorally to a titanium bonding base. The titanium support of the base gives the abutment complex improved mechanical properties [8]. The entire hybrid abutment is then screwed onto the implant, and, finally, the crown is cemented intraorally to this hybrid abutment (i.e., the ceramic abutment and the titanium bonding base). A hybrid abutment crown is a fully anatomical ceramic crown instead of an individual ceramic abutment, and it is bonded extraorally to the titanium bonding base. The hybrid abutment crown is finally screwed onto the oral implant intraorally. The crown margin usually is located below the soft tissue margin, and (in the case of bone-level implants) the crown per se forms the emergence profile. A major advantage of this method is that there is no need for intraoral cementation, which may play a role in terms of peri-implantitis prophylaxis. Both methods, using a hybrid abutment plus crown or a hybrid abutment crown, did not differ significantly in terms of fracture strength in an in vitro environment where a zirconium dioxide ceramic was used for the superstructure [12,13]. However, the workload and therefore the costs of the hybrid abutment crowns seemed to be lower, especially in conjunction with the digital workflow [14]. Initial clinical studies showed promising results for the hybrid abutment crown treatment option [15,16]. Takano and his team (2023) reported a fracture strength for the hybrid abutment crowns that exceeds the maximum masticatory force in the premolar region in a clinical situation [17]. However, long-term clinical experimental studies have not yet been conducted. In addition, the ceramic blocks used for the fabrication of these types of superstructures are delivered with prefabricated holes, which passively fit onto the prosthetic part of the titanium bonding base, enabling precise positioning of the restoration with anti-rotational features [18,19,20]. 

The attempt to connect the zirconium dioxide hybrid abutment crown to the corresponding titanium base without the use of additional cementation has shown inferior mechanical stability compared to the use of a cement layer in between the zirconium dioxide crown and the titanium base [21]. The authors concluded that the cement layer between the crown and the titanium base reduces the chances of abutment screw loosening and stress concentration on the implant–abutment complex and enhances the compressive load resistance [21]. For the luting of a crown onto a tooth or implant, conventional and adhesive cements have been widely used clinically [22]. In addition to their sealing effect and retention functions, the luting agents also assure a safe transfer of force from the restoration to the supporting abutment [23]. Conventional cements, e.g., zinc oxide phosphate and glass ionomer cements, have been applied in dentistry since the 1870s and have proven to be clinically reliable materials [24,25,26,27,28]. They mainly depend on macroscopic retention and micromechanical wedging of the prosthetic restoration [24]. On the other hand, adhesive cements provide a micromechanical–chemical bond between the two surfaces of the bonded materials and the cement [29,30]. Recently, the number of adhesive cements available on the market have increased [31]. For example, self-adhesive composites, which have special additives in their matrix, such as acidic methacrylate monomers with a phosphate group, seemed to be easier to use than conventional cements. The special additives eliminate pre-treatment steps of the materials to be bonded and make the application more user-friendly [31]. MDP (10-methacryloyloxydecyldihydrogenphosphate) is an acidic methacrylate monomer that is used in the bonding of high-strength oxide ceramics and non-precious alloys. With its acidic phosphate part, it chemically binds to the surface oxides of non-precious metals, titanium, and oxide ceramics through a condensation reaction. In addition, it contains a hydrophobic group with unsaturated methacrylates, which can form a chemical bond with the cementing composite. The use of reliable techniques for bonding zirconium dioxide abutment crowns to prefabricated titanium bases obviously improves the success rate of implant-supported crowns. Nevertheless, the connection of the zirconium dioxide hybrid abutment crown to the titanium bonding base needs to be further investigated in order to determine the safest method to maintain a durable and reliable connection, which type of cement should be used between both components, and which surface treatment of both titanium and zirconium dioxide can be recommended [32]. Therefore, the aim of this study was to examine the impact of the type of luting agent used between the zirconium dioxide hybrid abutment crown and the titanium bonding base on the retention and durability of the implant-abutment-prosthetic superstructure complex. 

Our null hypothesis was that there is no difference with regard to retention stability when using various luting agents to secure a zirconium dioxide hybrid abutment crown to a titanium bonding base after long-term chewing simulation and thermocycling.

## 2. Materials and Methods

**Figure 1:**
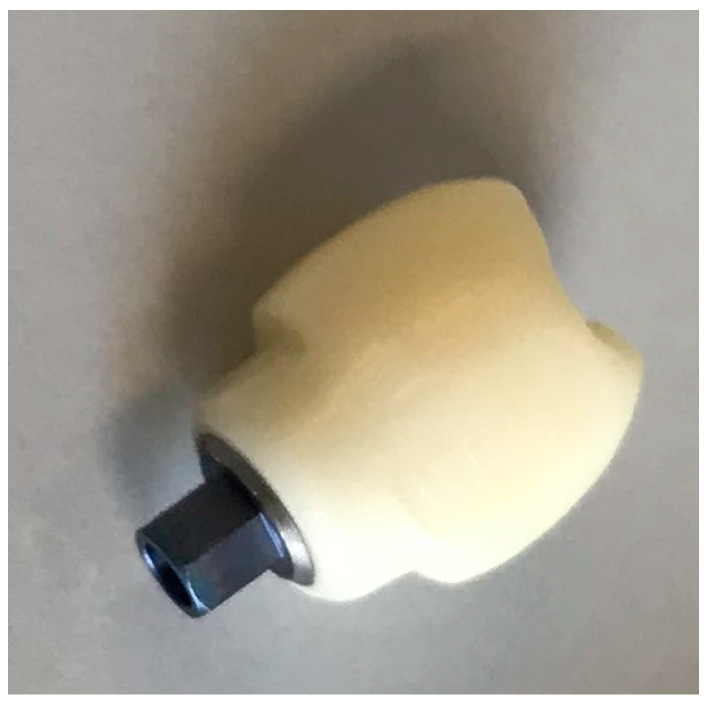
The hybrid abutment crown cemented onto the titanium bonding base.

Fifty-six monolithic zirconium dioxide hybrid abutment crowns (Sirona Dental Systems GmbH, Bensheim, Germany) bonded to their corresponding titanium (Grade 5) bonding bases (SIC Invent AG, Basle, Switzerland) were evaluated in this in vitro study (Figure 1).

The samples were divided into seven main groups according to the conventional or adhesive cement used to lute the zirconium dioxide hybrid abutment crown to the titanium base. The superstructures (the zirconium dioxide crown and the titanium base) were screwed into titanium (Grade 4) oral implants (SICmax^®^, SIC Invent AG) that were 14.5 mm long and had a diameter of 4.2 mm. 

**Figure 2:**
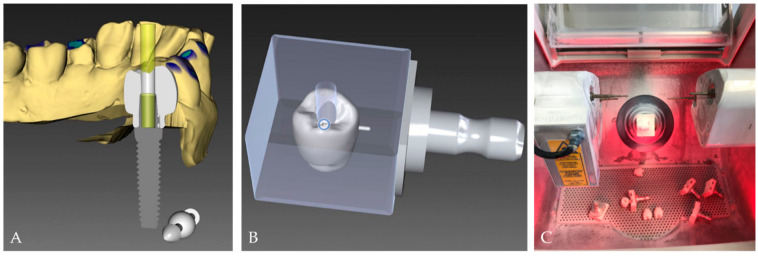
Computer-aided crown design steps (CAD) (**A**,**B**) and computer-aided crown manufacturing (CAM) (**C**) using inLab CAM software and an inLab MC XL milling machine. See the extended undercuts in (**A**) buccally and lingually for the pull-off testing.

The hybrid abutment crowns used in this study were fabricated from inCoris Zi meso blocks (Sirona Dental Systems GmbH, Bensheim, Germany). The chemical composition and the technical data of the zirconium dioxide blocks used to create the crowns are listed in Table 1 and Table 2. Simulating a clinical case, the crowns were designed as lower right second premolars using inLab CAM software (version 16.2, Dentsply Sirona, Charlotte, NC, USA) and milled using an inLab MC XL milling machine (Dentsply Sirona). Two (buccal and lingual) overhangs were designed at the cervical portion of the crown to allow for the performance of the pull-off test in a universal testing machine following thermomechanical fatiguing. After the milling process, the crowns were finally sintered according to the manufacturer’s instructions in a standardized process in an oven (VITA ZYRCOMAT 6000 MS, VITA Zahnfabrik, Bad Säckingen, Germany) to obtain their final strength and dimension (Figure 2). The inCoris blocks were featured with a ready-made connection geometry to passively fit to the titanium bonding base with a cementation gap determined by the manufacturer.

**Figure 3:**
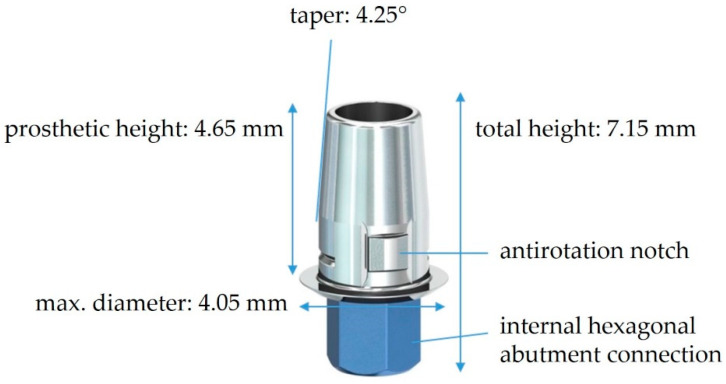
The specifications of the titanium bonding base straight CAD/CAM used in the current study (image source: https://shop.sic-invent.com/en, accessed on 16 November 2023).

Fifty-six prefabricated titanium bonding bases straight CAD/CAM (SIC Invent AG) were used to support the zirconium dioxide hybrid abutment crowns. The diameter of the titanium bases was 4.05 mm. All other dimensions are described in Figure 3. The titanium bases consist mainly of two parts: the prosthetic part to which the crown is cemented and the internal hexagonal abutment connection part that is retained to the implant with a screw. The prosthetic portion of the titanium base has a geometry that matches the connection geometry of the inCoris blocks. Thus, the dimension of the cement gap, as well as the anti-rotation protection notches, were determined by the manufacturer. 

For cementation, the following seven luting agents were used:

- SpeedCEM Plus (Ivoclar Vivadent AG, Schaan, Liechtenstein)
- Panavia SA Cement Universal (Kuraray Noritake Dental, Inc., Okayama, Japan)
- Panavia V5 (Kuraray Noritake Dental, Inc.)
- RelyX Unicem 2 Automix (3M Espe, Seefeld, Germany)
- VITA ADIVA IA-Cem (VITA Zahnfabrik, Bad Säckingen, Germany)
- Ketac CEM (Aplicap) (3M Espe, Seefeld, Germany)
- Hoffmann’s Phosphate Cement (Hoffman Dental Manufaktur GmbH, Berlin, Germany)

The intaglio bonding surface of the zirconium dioxide hybrid abutment crowns and the outer surface of the prosthetic part of the titanium bases were air-abraded using 50 µm Al_2_O_3_ particles (Pico-Edelcorund 50 µm, picodent^®^ Dental-Produktions- und Vertriebs-GmbH, Wipperfürth, Germany) and a laboratory blasting device (P-G 400, Harnisch + Rieth GmbH & CoKG, Winterbach, Germany). A distance of approximately 5–10 mm between the air abrasion nozzle and the specimen was maintained during the abrasion process. The blasting was performed for 30 s. The pressure used during the air abrasion was adjusted for each group according to the different manufacturers’ recommendations and the statement of EADT [33] (Table 3). After air abrasion, the titanium bonding bases and the zirconium dioxide hybrid abutment crowns were cleaned in an ultrasonic bath (Sonorex Digitec, Bandelin electronic GmbH & Co.KG, Berlin, Germany) with alcohol (Isopropanol 70, Brenntag GmbH, Essen, Germany) for three minutes and were then dried in an oil-free air stream.

The samples were divided into seven groups according to the cement used (Table 4) to bond the zirconium dioxide hybrid abutment crown to the titanium bonding base (for chemical composition see also Table 4).

The zirconium dioxide hybrid abutment crowns were cemented to the bonding bases according to the manufacturers’ recommendations for each luting agent group. The cements were processed according to the respective manufacturers’ instructions and applied to the surface of the bonding base. Then, the crown was manually cemented onto the abutment and fixed for one minute using gentle finger pressure. The complex of the zirconium dioxide hybrid abutment crown and the titanium base was then placed in a standardized loading apparatus with a constant vertical load of 7.4 N (750 g) for ten minutes during the setting phase of the cement. The excess cement was removed with foam pellets, and, where necessary, a glycerin gel was applied to prevent the formation of an oxygen inhibition layer. Light polymerization was not performed. For conventional cements, the excess cement was removed with a dental probe after setting. After the cementation process, the superstructures were each screwed into an embedded implant using titanium screws (Grade 5). The screws were 9.5 mm long, with a maximum diameter of 1.9 mm, and an M1.6 thread. They were tightened with a manual torque ratchet (SIC Invent AG) according to the manufacturer’s instructions with a torque of 20 Ncm. The occlusal access cavity of the crown was not sealed in order to allow for checking, retightening, or changing the screw in case of screw loosening during the dynamic loading.

**Figure 4:**
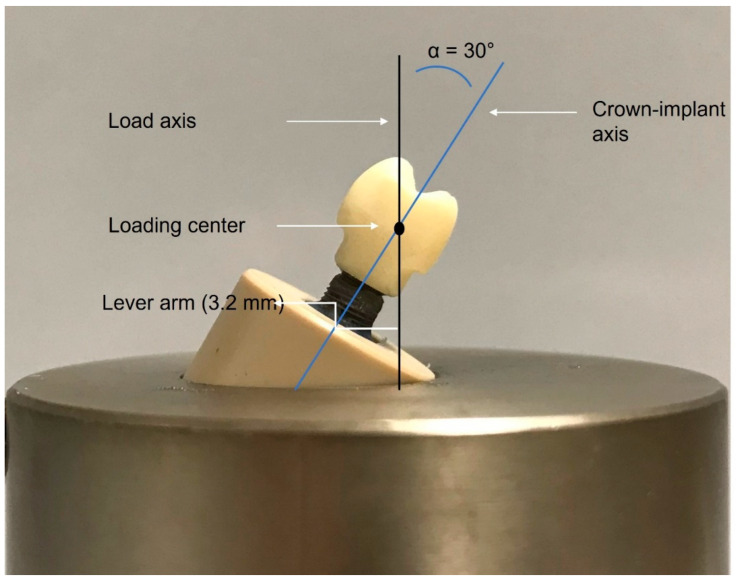
A photograph showing a lateral view of one sample before loading. The oblique blue line represents the long axis of the implant and the crown. The vertical black line represents the force direction in the chewing simulator.

In order to fix the implants with the crowns firmly and to ensure reproducibility in the chewing simulator, all implants were inserted in special polyether ether ketone (PEEK) tubes with an adjustable inner bottom. Following the ISO 14801 [34] requirements, 3 mm between the upper surface of the PEEK tube and the implant shoulder were left uncovered to simulate marginal bone loss. A prefabricated external fixation device was used to allow for the precise perpendicular embedding of all implants. A dual polymerizing acrylic resin (LuxaCore^®^ Z-Dual, DMG, Hamburg, Germany) was injected into the PEEK tubes around the implants for permanent embedding. The embedding material was intended to simulate bone that naturally surrounds the implant. The composite material had a modulus of elasticity greater than 3 GPa, which met the requirements of ISO 14801 [35]. The PEEK cylinders had a plate on the inside, on which the implant could be placed using the specific alignment device previously described [36]. To test the samples in the worst-case condition, all specimens were inserted into prefabricated aluminum cases so that the central longitudinal axis made a 30° ± 2° angle with the loading direction of the chewing simulator (Figure 4). 

To simulate the chewing masticatory forces and variations in temperature in the oral cavity, all samples (*n* = 56) were subjected to alternating thermal loads in a chewing simulator (Type CS-4.8, SD Mechatronik, Feldkirchen-Westerham, Germany). A total of five million mechanical cycles (reproduction of approximately 20 years of clinical scenario) [37] were conducted with thermocycling ageing (temperatures of 5 °C and 55 °C). The combination of horizontal movement (*X*-axis) (1 mm) and vertical movement (*Y*-axis) (2 mm) were applied to simulate the physiological chewing conditions with a cycle frequency of 1.2 Hz, resulting in a total load time of 48 days per test group.

**Figure 5:**
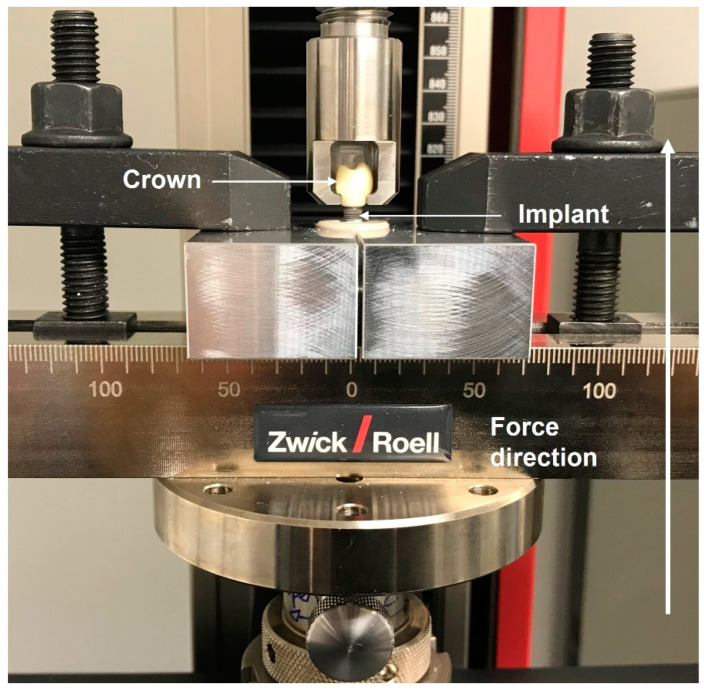
A test sample installed into a universal test machine using a special holder and a special jig for the bond strength testing procedure.

After the samples had been aged by chewing simulation and thermocycling, the pull-off forces were measured in a universal testing machine (Z2.5 ZwickRoell, ZwickRoell AG, Ulm, Germany). To carry out this part of the experiment, an individualized holder (Figure 5) was designed specifically for the purpose of gripping the crown at the two cervical notches. To firmly fix the abutment to the implant and PEEK cylinder, a customized device was also made in the form of two aluminum blocks. The pulling force was increased at a constant speed (1 mm/min) until the adhesive bond between the crown and the abutment came loose. The force of displacement (measured in N) was recorded for each test specimen using a specific software (testXpertIII, ZwickRoell AG, Ulm, Germany). The samples with decementation during the artificial thermocycling have been included in the statistical analysis with a pull-off force value of 0 N. After the specimens had been submitted to pull-off tests in the universal testing machine, they were examined to characterize the failure mode. The pull-off force was transferred to the shear bond strength in order to determine also the tensile strength. The debonding pull-off force measured in Newtons (N) was converted into Megapascals (MPa) using the following formula:

Shear bond strength = Pull-off force (N)/Cementation area (mm^2^) (1)

where, the cementation area of the titanium bonding base used in this study was 50.5 mm^2^.

After the pull-off test, each sample was examined visually using magnifying dental loups (head magnifier KF, Carl Zeiss AG, Oberkochen, Germany) with 3.3× magnification and additional LED lighting, as well as a dental probe to assess the failure pattern of the cement after the pull-off test. Each sample was assigned to one of the five following categories according to the distribution of cement residues: 

Category A: Predominant or complete adhesive failure at the ZrO_2_ interface: the adhesive titanium base is covered with a visible cement film; the inner part of the zirconium dioxide crown is not.

Category B: Predominant or complete adhesive failure at the adhesive titanium base interface: the inner part of the zirconium dioxide crown is covered with a visible cement film; the titanium base is not.

Category C: Predominant or complete cohesive failure: the adhesive titanium bonding base and the inner part of the zirconium dioxide crown are covered with a visible film of cement.

Category D: Predominant or complete mixed failure: the surfaces of both the titanium bonding base and the zirconium dioxide crown are partially exposed or not covered with cement; other areas are still partially covered with cement; opposite surfaces are not simultaneously covered with cement.

Category E: Minimal/hardly any cement overall: hardly any of the zirconium dioxide and titanium surfaces are covered with a visible film of cement.

The statistical analysis included the calculation of the mean values and standard deviations for the descriptive analysis of the values. In addition, boxplots were generated for graphical representation. One-way analysis of variance (ANOVA) was carried out to test for differences in pull-off force between the different cement groups investigated in this study. Furthermore, in subsequent pairwise comparisons, the Scheffe method was used to correct for multiple testing. The software STATA 17.0 (StataCorp, College Station, TX, USA) was used all data evaluations. The level of statistical significance was set at 0.05.

## 3. Results

After conducting the thermomechanical tests on the various groups, one sample showed both an abutment and a screw fracture (group 4 “RelyX Unicem 2 Automix”) after 2,778,775 chewing cycles. This sample was excluded from the statistical analysis. No other crown, abutment, screw, or implant fractures were observed, and no screw loosening or ceramic chipping was noted. This resulted in an overall implant and superstructure survival rate of 98.2%. Moreover, two samples in group 7 (Hoffmann’s phosphate cement) showed decementation after artificial thermocycling (before a pull-off test could be performed). Hence, a total of three samples showed complications (2× loss of retention, 1× abutment/screw fracture), which yielded a 5.4% rate of complications.

**Figure 6:**
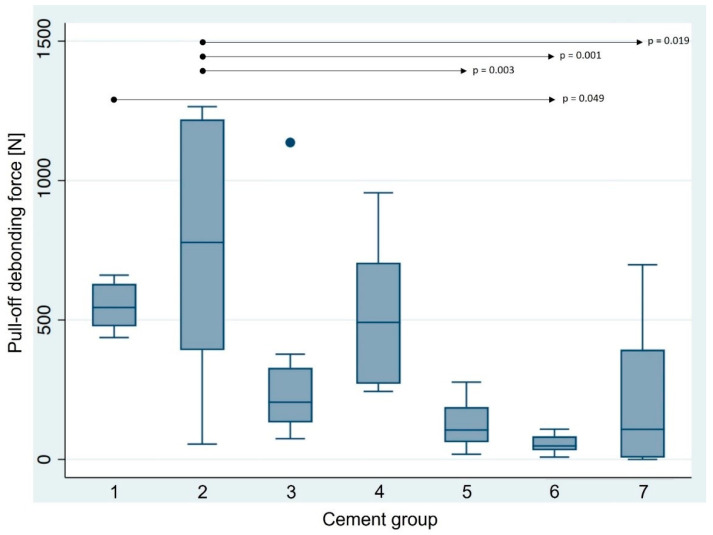
The boxplot diagram represents the bond strength values of the experimental groups (N = 55). Horizontal black lines indicate statistically significant differences among the seven groups. Group 1: SpeedCEM Plus; group 2: Panavia SA Cement Universal; group 3: Panavia V5; group 4: RelyX Unicem 2 Automix; group 5: VITA ADIVA IA-Cem; group 6: Ketac CEM; group 7: Hoffmann’s Phosphate Cement.

The debonding forces of the various luting agent groups measured in the pull-off test were between 0 N and 1265 N (Figure 6, Table 5). Group 6 [Ketac CEM (Aplicap)] exhibited the lowest pull-off debonding force with a mean value of 55 N, whereas group 2 (Panavia SA Cement Universal) had the highest retention value of 762 N. 

The evaluation of the pull-off debonding force using different luting agents revealed a statistically significant difference between the seven groups (*p* < 0.001). Group 1 showed a significant higher pull-off force compared to group 6 (*p* = 0.049). There were also significant differences between group 2 and groups 5 (*p* = 0.003), 6 (*p* = 0.001), and 7 (*p* = 0.019). All other pairwise comparisons did not show statistically significant differences (Figure 6).

**Figure 7:**
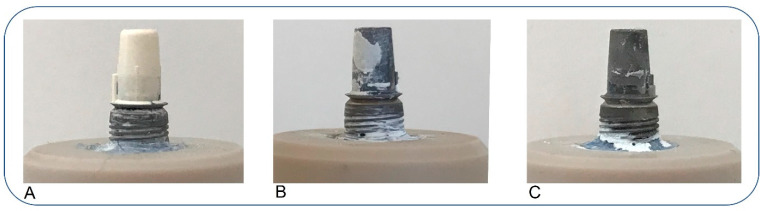
(**A**) A sample from group 5 showing an adhesive failure at the zirconium dioxide surface and the luting agent: the titanium bonding base is entirely covered with the luting agent (category A); (**B**) A sample from group 7 shows a mixed failure pattern: the titanium bonding base is partially covered with the luting agent (category D); (**C**) A sample from group 1 displays a failure pattern between the titanium bonding base and the luting agent: the bonding base does not have residues of the luting agent (category B).

The analysis of failure patterns showed predominant an adhesive failure pattern at the ZrO_2_ interfaces in group 4 and 5. In general, adhesive failures at the interface to the ZrO_2_ were dominant (38%). Adhesive failures between the titanium bonding base and the cement were detected in 9 cases (16%). Except for two samples in group 2, no further specimen experienced a cohesive failure (4%) (Table 6). For illustration, Figure 7 shows three abutments with an exemplary distribution of the failure patterns and cement residues. 

## 4. Discussion

The aim of this laboratory study was to evaluate the retention stability between the zirconium dioxide hybrid abutment crown and the titanium bonding base supported by a hexagonal internal implant–abutment connection when different luting agents are used. According to our results, the null hypothesis has to be rejected since the type of the luting agent significantly influenced the bonding strength. The selection of the different luting agents in this study was a critical aspect aimed at ensuring a comprehensive and meaningful comparison of different cement types, including self-adhesive, adhesive and conventional cements. Our criteria for selecting these luting agents were considered to address the primary objectives of the research and enhance the applicability of our findings according to the following factors: clinical relevance, literature review, material characteristics, application versatility, practical considerations, and reproducibility.

In our study, all samples were subjected to dynamic loading in an artificial chewing simulator before testing the retention force of the examined luting agents. It has been assumed that laboratory thermocycling ageing associated with dynamic fatigue could help inform about mechanical behavior and material properties of the tested specimens and additionally yield supportive information on how well or poorly certain materials withstand masticatory forces [38,39,40]. Moreover, since the adhesive cements are brittle materials, consecutive static loading until failure, in the form of tensile force, is considered to be an invaluable tool when comparing different systems [29].

In this study, one sample (1.8%) showed both an abutment and a screw fracture during the dynamic loading. This mechanical complication might have occurred as a consequence of undetected screw loosening [41]. Abutment screw loosening happens frequently in dental clinics, with reported rates between 2.25% and 11%, while abutment screw fractures are less prevalent and were found to occur in 0.6% of the cases [1,42]. Therefore, abutment screw loosening and fractures are some of the most serious and prevalent problems associated with restoring oral implants. The implant–abutment connection geometry (e.g., external vs. internal) and/or the force of the occlusal load (especially in the chewing simulation scenarios) might be influencing factors for screw loosening. As a consequence, this can lead to a micromovement of the supported structures and subsequently result in a screw fracture [43]. 

In addition to abutment and screw fractures, failures during chewing simulation included debonding between the zirconium dioxide hybrid abutment crowns and the titanium bonding bases in two of the samples (25%) in one cement group (Hoffmann’s phosphate cement) by either dissolution or leaching of the luting agent. In a previous in vitro study that investigated specimens in distilled water and artificial saliva, zinc phosphate cement was reported as having the highest rate of dissolution (compared to Rely X lute2, zinc polycarboxylate cement, Rely X U-200, and glass ionomer cement) [44]. In our study, ageing the samples in a wet environment, i.e., thermocycling, might have led to the dissolution of the zinc phosphate cement due to water absorption [45]. However, these complications can be repaired clinically by recementing the different parts extraorally. 

In previous studies, the bonding strength between zirconium dioxide superstructures and titanium bonding bases were evaluated in in vitro environments. Pull-off strengths between 147 N and 925 N [46,47,48,49,50,51,52,53] were reported. Furthermore, another study recommended that for the bonding strength of the luting agent to be considered safe for the clinical use, it should exceed the maximum extrusive force due to the contraction of mandibular depressor muscles during mastication (159 N) [54]. In our investigation, mean bonding strengths ranging between 55 N and 762 N were obtained when testing two conventional cements, two adhesive resin cements, and three self-adhesive resin cements after ageing and fatigue testing. Our results are therefore comparable to those of other investigations [46,47,48,49,50,51,52,53,54]. Moreover, most of the luting agents used in the current investigation are appropriate for bonding of zirconium dioxide crowns onto titanium bases since the debonding forces were above the recommended value (159 N) [54].

Conventional cements (glass ionomer cement and zinc phosphate cement) were used as control groups in the current investigation. Conventional cements, however, rely mainly on micromechanical interlocking factors (e.g., on height and the taper of the substructure), and it was emphasized that they might be suitable for a semipermanent cementation, which provides a predictable retrievability of implant-supported restorations [55,56]. In our study, mean bonding strengths of 55 N and 214 N were reported after pull-off tests were performed for the glass ionomer and zinc phosphate cement groups, respectively. The observed superior performance of zinc phosphate cements in our study may be attributed to their high elastic modulus. Zinc phosphate cements are characterized by a high elastic modulus, which is indicative of their ability to resist deformation under stress. This mechanical property suggests that zinc phosphate cements exhibit enhanced stiffness and rigidity compared to glass ionomer cements. However, these values were much lower than the pull-off bonding strength values reported in a previous study [57]. Schiessl et al. [57] investigated the bonding strength between zirconium dioxide crowns and titanium abutments using different luting agents, including glass ionomer and zinc oxide phosphate cements, after thermal artificial ageing (6000 thermocycles (5 °C/55 °C) without a chewing simulation). The authors reported mean pull-off bonding strengths of approximately 240 N and 330 N for the glass ionomer and zinc phosphate cements, respectively. The difference between our results and the former are potentially due to the different study designs given that dynamic loading was not applied in the study by Schiessl et al. It was assumed that the stress caused by dynamic loading in a wet environment led to disintegration and leaching of the luting agent, which led to the weakening of the cement interfaces with the bonded surfaces [58]. Furthermore, the setting of the cement in the present study occurred in a dry environment, while Schiessl et al. stored the specimens in water for 24 h 10 min after the cementation procedure. Both of the above factors could have led to the reduced retention values of the present study [44].

The bonding strength between the zirconium dioxide hybrid abutment crown and titanium bonding base was evaluated in the current study using two adhesive cements (Panavia V5; VITA ADIVA IA-Cem). In the group where the specimens were cemented with Panavia V5, a mean bonding strength of 318 N (shear strength: 6 MPa) was reported without a significant difference compared to any of the other cements used in this study. In a prior investigation, the combination of Panavia V5 and Clearfil primer was reported to operate effectively on both polished and sandblasted zirconia surfaces, yielding consistently elevated shear bond strength values [59]. Mean bonding strength values of 428–804 N [46] and 126 N [60] were reported in previous studies when Panavia V5 was used for the cementation procedure between zirconium dioxide crowns and titanium abutments/bonding bases. Arce and coworkers investigated the retention strengths between zirconium dioxide crowns and titanium bases (containing retentive grooves) following different surface treatments before bonding the different parts with Panavia V5 adhesive cement. The authors reported a mean bonding strength of 596 N when applying the surface treatment, which was identical to the one applied in our study, i.e., alumina airborne-particle abrasion and a MDP primer [46]. A possible explanation for the lower retention values reported in the current study for the Panavia V5 group compared to values reported by Arce et al. may be attributed to the long-term artificial loading performed in this study [60]. 

To the knowledge of the present authors, there are no data available regarding the bonding strength of the adhesive cement VITA ADIVA IA-Cem, which was used in this study. However, the bonding strength value of 126 N obtained in our study for this cement group was lower than the retention values obtained with Panavia V5 and the self-adhesive cements used in the current investigation. Further laboratory investigations should be performed with this luting agent in order to provide more pre-clinical information about whether this retention force is sufficient for long-term clinical use.

Self-adhesive cements (SpeedCEM Plus, Panavia SA Cement Universal, RelyX Unicem 2 Automix) investigated in the current study showed the highest bond strengths ranging between 508 N and 762 N. No statistically significant differences in the pull-off bonding strengths between the different self-adhesive cements were observed after ageing and loading were applied to all test groups. This result is in contrast to those reported in an in vitro study by Mehl et al. [48]. The latter authors found significant differences in the bonding strengths when using the following self-adhesive cements: Panavia SA Cement Automix, RelyX Unicem 2 Automix, MaxCem Elite, and SmartCem 2. Mean bonding strengths ranging between 350 N and 887 N were reported in the study by Mehl et al., and these values differed from those obtained for the self-adhesive cements (SpeedCEM Plus, Panavia SA Cement Universal, and RelyX Unicem 2 Automix) used in our study (508–762 N). Variations between the two studies may be explained by differences in the study protocols given that dynamic loading was not applied before the pull-off test in the study by Mehl et al. (2018). It has to be assumed that an extended period of artificial ageing negatively affected the properties of the dental cements and, therefore, the bond strength [47,61]. When directly comparing the same self-adhesive cements used in both studies, Mehl et al. showed that the groups cemented with Panavia SA and RelyX Unicem 2 Automix had the highest bond strength (688 N for Panavia SA adhesive cement and 887 N for RelyX Unicem 2 Automix adhesive cement), and these values are similar to the values obtained in our investigation. Based on the results of both studies, prolonged dynamic loading seems to have no influence on the bonding strength of the two cements, namely, Panavia SA and RelyX Unicem 2 Automix. Panavia SA self-adhesive cement, which presented the highest bond strength (762 N) in the current study, is characterized by a larger grain size and contains a long-chain silane (LCsi) that offers enhanced surface area and potentially contributes to improved bonding strength [62]. 

The different luting agents used in the current investigation showed different fracture patterns after the pull-off test. Adhesive failures at the interface to the ZrO_2_ were frequently seen (38%) compared to the adhesive fracture pattern observed between the titanium bonding base surface and the luting agent (16%). This demonstrates that the bonding between the zirconium dioxide surface and the luting agent is the weakest point of assembly. Our results are in agreement with previous studies, reporting that the ZrO_2_/adhesive interface is typically the most fragile point of the bond system [47,48,51,63,64]. A possible reason for the weaker bond between the adhesive cement/primer and the zirconium dioxide surface compared to titanium may be the changes in the surface characteristics, i.e., an increase in the surface roughness and a decrease in the hardness caused by the low-temperature degradation at the zirconium dioxide interface [65,66]. However, this explanation needs to be confirmed in further investigations. In the current investigation, cohesive fractures, which occurred within the luting agent layer, were only observed in two samples of the Panavia SA self-adhesive resin cement group. Interestingly, this group showed the highest shear bond strength (15 MPa) compared to the other groups tested in our study. These findings are in agreement with previous studies, where higher cement retention values were obtained for the groups that presented cohesive fracture patterns in some of the tested samples [63]. 

The technical parameters used in in vitro studies might provide pre-clinical data about the mechanical behavior and the material properties of the investigated specimens along with supportive information on material deficiencies which may arise due to masticatory forces. A direct transfer of the present data to the clinical situation is—as usual—limited. As mentioned by Güngör et al. (2018), the pull-off forces applied during the bonding shear strength test are unidirectional and do not represent/mimic the nonaxial occlusal forces in a patient’s mouth [47]. Moreover, when interpreting the findings of the current study, it is crucial to mention the limitation imposed by the relatively small sample size. Applying our data to an in vivo situation should be done cautiously. Additional clinical studies are required to provide more valuable data about the clinical reliability of the investigated cement systems used in our in vitro study.

## 5. Conclusions

Within the limitations of this study, the following conclusions can be drawn:

- Most of the luting agents tested in the current investigation are suitable for “cementation” of a zirconium dioxide hybrid abutment crown onto a titanium bonding base since the debonding forces are above a recommended value (159 N).
- The use of self-adhesive cements yielded the highest bonding strength for bonding a zirconium dioxide hybrid abutment crown onto a titanium bonding base after long-term artificial fatigue and aging.
- The adhesive bond to zirconium dioxide is more vulnerable than the adhesive bond to titanium.

## Figures and Tables

**Table 1:** Chemical composition of the zirconium dioxide inCoris blocks used for crown fabrication.

Components	Amount (in %)
ZrO_2_ + HfO_2_ + Y_2_O_3_	≥99.0
Y_2_O_3_	>4.5–≤ 6.0
HfO_2_	≤5
Al_2_O_3_	≤0.5
Fe_2_O_3_	≤0.3

**Table 2:** Technical data of the zirconium dioxide inCoris blocks.

Density	6.05 ± 0.2 g cm^−3^
Fracture toughness KIC	5.8 MPa m^1/2^
Thermal expansion coefficient (20–500 °C)	11 × 10^−6^ K^−1^
Bending strength	1200 MPa

**Table 3:** Air abrasion pressures for the different cements.

Cement	Air Abrasion Pressure
SpeedCEM Plus	2 bar
Panavia SA Cement Universal	2 bar
Panavia V5	2 bar
RelyX Unicem 2 Automix	2 bar
VITA ADIVA IA-Cem	1.5 bar
Ketac CEM (Aplicap)	1 bar
Hoffmann’s Phosphate Cement	1 bar

**Table 4:** Luting agents used in this study and the material composition provided by the manufacturers.

Group (Number of Samples)	Cement Type	Type and Chemical Components	Special Features, Preparation, Processing
Group 1 (*n* = 8)	SpeedCEM Plus	Resin cement: long-chain methacrylate with a phosphoric acid monomer (MDP), barium glass, ytterbium trifluoride, co-polymer, and highly dispersed silicon dioxide	Dual curing (self-curing with option to light cure) Primer: usable with/without Monobond Plus (in this study, Monobond was applied only to the titanium bonding base) Self-adhesive Double-push syringe delivery form
Group 2 (*n* = 8)	Panavia SA Cement Universal	Resin cement: 10-methacryloyloxydecyl dihydrogen phosphate (MDP), bisphenol A diglycidylmethacrylate (Bis-GMA), triethyleneglycol dimethacrylate (TEGDMA), hydrophobic aromatic dimethacrylate, 2-hydroxymethacrylate (HEMA), silanated barium glass filler, silanated colloidal silica, dl-camphorquinone, peroxide, catalysts, and pigments	Dual curing (self-curing with option to light cure) Self-adhesive Double-push syringe delivery form
Group 3 (*n* = 8)	Panavia V5	Resin cement: Paste A: bisphenol A diglycidylmethacrylate (Bis-GMA), triethyleneglycol dimethacrylate (TEGDMA), hydrophobic aromatic dimethacrylate, hydrophilic aliphatic dimethacrylate, initiators, accelerators, silanated barium glass filler, silanated fluoroalminosilicate glass filler, and colloidal silica Paste B: bisphenol A diglycidylmethacrylate (Bis-GMA), hydrophobic aromatic dimethacrylate, hydrophilic aliphatic dimethacrylate, silanated barium glass filler, silanated aluminum oxide filler, accelerators, dl-camphorquinone, and pigments	Dual curing (self-curing with option to light cure) Primer: Clearfil Ceramic Primer Plus (MDP) for both the zirconium dioxide crown and the titanium bonding base Double-push syringe delivery form
Group 4 (*n* = 8)	RelyX Unicem 2 Automix	Resin cement: Base paste: methacrylate monomers containing phosphoric acid groups, methacrylate monomers, silanated fillers, initiator components, stabilizers, and rheological additives Catalyst paste: methacrylate monomers, alkaline (basic) fillers, silanated fillers, stabilizers, pigments, and rheological additives	Dual curing (self-curing with option to light cure) Self-adhesive Double-push syringe delivery form
Group 5 (*n* = 8)	VITA ADIVA IA-Cem	Ultra-opaque resin cement: mixture of resin based on Bis-GMA, catalyst, stabilizer, and pigments	Dual curing (self-curing with option to light cure) Primer: VITA ADIVA ZR-Prime for the zirconium dioxide crown, Monobond Plus for the titanium bonding base Double-push syringe delivery form
Group 6 (control) (*n* = 8)	Ketac CEM (Aplicap)	Glass ionomer cement: Powder: glass powder (CaAlFsilicate) and pigments Liquid: polycarboxylic acid, tartaric acid, water, and conservation agents	Self-curing (chemical) Mixing capsule delivery form
Group 7 (control) (*n* = 8)	Hoffmann‘s Phosphate Cement	Zinc phosphate cement: Powder: zinc oxide and magnesium oxide Liquid: ortho-phosphoric acid	Self-curing (chemical) Manual mixing of the powder and the liquid

**Table 5:** Descriptive statistical analysis of the pull-off test values (N) and the shear bond strength (MPa) of the different groups.

Group (n Samples)	Mean	Standard Deviation	Minimum	Maximum	Shear Bond Strength
1 (8)	550 N	85 N	437 N	661 N	11 MPa
2 (8)	762 N	476 N	55 N	1265 N	15 MPa
3 (8)	318 N	345 N	74 N	1136 N	6 MPa
4 (7) ^a^	508 N	255 N	244 N	956 N	10 MPa
5 (8)	126 N	86 N	18 N	277 N	3 MPa
6 (8)	55 N	34 N	8 N	108 N	1 MPa
7 (8)	214 N	259 N	0 N ^b^	698 N	4 MPa

^a^ One sample did not survive the thermocycling. ^b^ Some samples debonded (loss of retention) before the pull-off test.

**Table 6:** The results of adhesive/cohesive failure modes of the experimental groups.

Category	A	B	C	D	E
Group 1	0	5	0	3	0
Group 2	1	2	2	3	0
Group 3	5	0	0	3	0
Group 4	7	0	0	0	0
Group 5	8	0	0	0	0
Group 6	0	0	0	6	2
Group 7	0	2	0	4	2

Category A: The titanium bonding base is covered with a visible cement film; the inner part of the zirconium dioxide crown is not. Category B: The inner part of the zirconium dioxide crown is covered with a visible cement film; the titanium base is not. Category C: The titanium bonding base and the inner part of the zirconium dioxide crown are covered with a visible film of cement. Category D: The surfaces of both the titanium bonding base and the zirconium dioxide crown are partially exposed or not. Category E: Hardly any of the zirconium dioxide and titanium surfaces are covered with a visible film of cement.

## Data Availability

Data are contained within the article.
